# Unconventional Practices and Boundary Work in Small Town Newspapers: The Case for a Rural Journalism Subfield

**DOI:** 10.1177/10776990251407086

**Published:** 2026-01-30

**Authors:** Tyler W. S. Nagel, Marcel Broersma

**Affiliations:** 1 Centre for Media and Journalism Studies, University of Groningen, the Netherlands; 2 Southern Alberta Institute of Technology, Calgary, Canada

**Keywords:** local journalism, rural journalism, field theory, boundary work, print journalism

## Abstract

Local rural newspapers live a precarious existence in Alberta, Canada. In a declining economic situation, rural journalists have adopted unconventional practices to continue their work. Based on fieldwork in four communities, we show how rural journalists negotiate between the dominant metropolitan-based doxa and habitus of the journalistic field and their own position within communities. The analysis reveals rural-specific reporting practices, atypical business practices, and unconventional approaches to innovation. We argue that rural journalism is not merely a deviation from dominant metropolitan norms but a subfield actively redefining the boundaries of journalistic practice in response to economic precarity and community obligations.

It’s a rainy day in a remote Canadian town of 3,000 people, and not the weather for some large-caliber rifle practice at a gun range. But the local newspaper reporter, decked out in rain gear and keen to show the best of their small town, insists on braving kilometers of dubious muddy logging road to show the beautifully maintained shooting facility carved out of the dense forest. Community pride is evident during the tour of the volunteer-built clubhouse, shooting stands, trap-shooting area, long-distance ranges, and safe gun-handling policies. At the end of it, the local reporter excuses themself and drives their well-worn pickup truck back to town: they must get ready for a town council meeting. They are attending not as a member of the press—but as an elected town councilor. At the meeting, the creation of a new road-worker position is discussed in detail and eventually unanimously approved by the council (including the reporter). Later that evening, after the meeting ended, they wrote about it for this week’s issue of the newspaper, using objective wording. They are proud of the paper, explaining it’s the only source of news available to the townspeople. There is no local news radio, and no local television news. The big-city Canadian daily newspapers are no longer shipped to this town. And the internet connection that delivers national news is often slow. This news reporter cum politician serves a vital role in their news-starved community.

Such arrangements, unfamiliar as they may seem to journalists and journalism scholars accustomed to major-market newspapers in Western countries, are common in today’s rural publications. Beyond political involvement by reporters, other forms of rural reporting practices (such as allowing sources to vet stories prior to publication, uncommon in North American newspaper journalism), business practices (distribution deals with local government), and news values (running good-news stories only) differ from dominant metropolitan journalistic doxa and habitus.

Differences between rural and urban news have long existed (Nielsen, 2015). In small rural towns in the Canadian province of Alberta, running a newspaper has always been a difficult undertaking. When the area was settled only about 130 years ago, newspaper owners came across the bald prairie with a printing press in the back of a horse-drawn wagon or loaded on a railway boxcar. The owner *was* the publication. There was no “ethical wall” separating editorial and business functions of the newspaper because the owner-publisher-editor-advertising sales rep-press operator did it all. Even in those pioneer times, journalistic practices and ethics that were already conventional at large urban publications weren’t easily applicable to the conditions in which these small newspapers operated (Allen, 1928). Like today’s entrepreneurial start-ups (Broersma & Singer, 2021; Hess & Waller, 2016), such business ethics were a luxury that could not always be afforded by the local newspaper owner in a tiny prairie town. Naturally, practices have evolved since early pioneer days. But, as described in the gun range vignette above, there remains considerable divergence between practices of rural journalists and the norms of journalists working in metropolitan areas. To understand this anomaly, we draw on Bourdieu’s (1983) field theory, which describes dominant doxa and habitus as defining elements of a particular field. In the context of this divergence of rural journalism from established urban journalistic practices, this study explores how rural journalists negotiate between the dominant metropolitan-based doxa and habitus of the field and their position within rural communities.

The Canadian province of Alberta was chosen as the research site. Outside its major metropolitan corridor, rural towns are geographically separated in Alberta with diverse economies and a variety of newspaper ownership structures, providing an ideal location to examine a range of rural news organizations. Grounded in in-person community visits and interviews with journalists, publishers and community members, our analysis shows a complicated situation where field norms are broken. Rural newspapers are pulled between the cultural practice of “pure” journalism, rural community audience expectations, and the economic exigencies of running rural newspapers in a declining fiscal situation.

## Rural Journalism

Rural journalism is a sub-practice of local journalism that, despite the increasing interest in local journalism research (Gulyas et al., 2023), has received little attention from scholars so far (Örnebring et al., 2020; Wenzel, 2018). Partially stymying these efforts is that the term rural is ill-defined, and the term rural journalism is a contested term among journalism scholars (Ferrucci, 2024; Mathews, 2022). We assume a conventional understanding of the term rural, as defined by three factors: small scale, low density settlement; distance from urban centers; and specialization of economies (Deavers, 1992). But no matter the academic definitions of rural, the people who live in rural areas view themselves as distinct from urbanity, often viewing urban hegemonies with animosity (Cramer, 2016).

When considering journalism that happens in such rural areas, a particular concern is the differentiation of the terms rural, local, and community journalism (Ferrucci, 2024). Generally, local journalism describes the proximity of the journalist to the audience, and community journalism describes a specific practice that emphasizes coverage relating to daily life and an intimate connection to the community (Ferrucci, 2024; Lauterer, 2006). However, local and community journalism are overlapping terms (Gulyas & Baines, 2020). Each can occur in urban areas as well as rural ones. But journalism in rural areas is different than major-market journalism, and such differences have long been recognized (Abernathy, 2016; Allen, 1928; Nagel, 2015). Such variation from major market journalism means that rural journalists “face their own challenges and opportunities” (Ali et al., 2020, p. 454).

Distance, both between a newspaper and audiences, as well as distance between a given rural community and neighboring urban areas, is an important factor when examining differentiating aspects of rural journalism (Freeman, 2020). But as discussed above, distance is not strictly a geographic conceptualization. “Community newspapers have something that city dailies lack—nearness to people” (Byerly, 1961, p. 25). Such social intimacy with the community is a hallmark of rural journalism that simultaneously reinforces the role of the rural journalist to provide an unbiased record of happenings to the community and yet be a model community member (Allen, 1928). Tensions, then, play out in the expected rural journalistic performance, such as community boosterism. This is sometimes accompanied by misgivings from the journalists themselves about the compromises necessary to fulfill both the role of community member and of journalist (Perreault et al., 2023). As this paper will discuss, the role of the journalist in the introduction—simultaneously objective community reporter and engaged local politician—exemplifies such dual allegiances in rural journalism roles.

But despite efforts to define the location and practices of rural journalism, it is not homogenous, especially when considering Indigenous and non-North American contexts. Conceptualizations of the terms rural, journalism, and rural journalism itself vary considerably when compared against North American understandings (Baines, 2013; González De Bustamante & Relly, 2016; Henrichsen, 2022; Humprecht et al., 2022).

## A Field Perspective on Rural Journalism

Increasingly, there is uncertainty in what, exactly, the boundaries of journalism are (Eldridge, 2022). In exploring the delineation of journalism boundaries in rural newspapers, we draw on Bourdieu’s field theory. Bourdieu posits that society can be divided into various social fields that may be distinct, overlapping, or nested like Russian dolls (Fligstein & McAdam, 2012). Each field represents a distinct area of social life, such as science, art, or politics. Essentially, they are constellations of forces in which various agents strive for power. Fields are structured by these relations and have their own rules, hierarchies, and power dynamics (Bourdieu, 1993). Agents within a field aim to gain economic, cultural, and symbolic capital to distinguish themselves from others, obtain more power, and improve their position in the field.

A field, such as journalism, is guided by its *doxa*, the “tacit presuppositions that we accept as natives of a certain society” (Bourdieu, 2005, p. 37), which are often taken for granted and difficult to make explicit. Within journalistic doxa exist norms. In many cases, such norms are learned intrinsically from other actors in the newsroom (journalists or editors) but can also be formally learned in journalism schools. They are also codified in association industry codes of ethics, for example, the Canadian Association of Journalists (2011) ethics guidelines, and journalism guidebooks have been written about them—the canonical *Elements of Journalism* among the most prominent (Kovach & Rosenstiel, 2021). Another central concept in field theory is *habitus*, “the capacity to produce classifiable practices and works, and the capacity to differentiate and appreciate these practices” (Bourdieu, 1984, p. 170). Habitus stems from long-term socialization, including upbringing and professional training (Benson & Neveu, 2005). In the case of the journalism field, doxa (through its association with journalistic norms) and habitus inculcate journalistic practices in field members.

Within cultural fields, Bourdieu (1983) conceived of an “autonomous” pole and a “heteronomous” one. The autonomous pole is where laws (nomos) are controlled from within the field. The heteronomous pole is where external forces guide the laws of the field—with economic forces among the most prominent. In the larger field of cultural production, journalism tends to be pulled more toward the heteronomous pole than the autonomous one by virtue of its reliance on advertising, subsidy, and subscription (Bourdieu, 2005). Countering the pull toward the heteronomous pole is the impetus to adhere to the ethics of the journalism profession, which are often pitted against economic realities of the newspaper business (Fengler & Russ-Mohl, 2008). But regardless of the position of the overall journalism field, actors within the field are also caught up in the tensions between the poles and are situated differently within the continuum between them. In this way, the boundaries of the journalistic field create a microcosm through which individual publications and even journalists may be understood (Benson & Neveu, 2005).

## Rural Journalism and Boundary Work

But what are the boundaries of the journalism field? Unlike professions such as engineering and medicine, there are few legislative dicta that define Western journalism (Carlson, 2015). Autonomously defined norms have enabled journalists to produce trusted work, free from external oversight (Singer, 2015). This internally defined yet vague normative ideal makes determining the boundaries of journalism difficult. This is exacerbated by a field that is rapidly changing in technological, economic, and social dimensions (Tandoc, 2019). Ryfe (2012) describes the field as unraveling—forces that formerly were cohesive now pull the practice of journalism apart. As well as the increasing prominence of quasi-journalistic interlopers (particularly online), which blur the line of what, exactly, journalism is, journalists themselves perform activities that challenge traditional conceptualization of North American journalistic practices. For example, Indigenous reporters, in hybrid community member/journalist roles, are sometimes hesitant to report on their home communities for fear of exclusion by said communities (Henrichsen, 2022). These reporters felt a stronger impetus to emphasize community cohesion over information provision. Such tensions between journalistic professionalism and societal constraints are evident in other contexts as well, such as reporting in countries with increased danger for journalists. Journalists must make compromises between their journalistic work and factors such as personal harm and the safety of their own families (González De Bustamante & Relly, 2016).

In a study of boundaries in the scientific community, Gieryn (1999) proposes three key types of boundary work: expansion, expulsion and protection of autonomy (Table 1). These activities are performed by the scientific community to delineate science from non-science and to preserve scientific authority. Extending those concepts to journalism, Carlson (2015, p. 9) juxtaposes Gieryn’s three areas of boundary work with the “areas of journalism around which boundary work occurs”: participants, practices, and protection of autonomy.

**Table 1.** Forms of Boundary Work in Journalism.

	Participants	Practices	Professionalism
Expansion	Incorporating non-traditional journalists	Taking over new media practices as acceptable	Absorbing new media as acceptable journalism
Expulsion	Expelling deviant actors	Expelling deviant practices	Expelling deviant forms and values
Protection of Autonomy	Keeping out non-journalistic informational actors	Defense of the ability to define correct practices	Defense from non-professional outsiders

*Source.* From Carlson (2015).

These areas are particularly important in the case of journalists, which, like architects, translators, body piercers, and pet sitters, do not require specific credentials to lay claim to their title (Lewis, 2012, p. 843; Tandoc, 2018). This is not new: historically, many journalists have had no formal journalism training (Wright, 1976). Absence of formal certification means that, in large part, the doxa of the journalism field defines the profession. Like other fields proposed by Bourdieu (2005, p. 39), differentiation of journalism from other associated fields is key to its existence. Nowadays, the journalistic struggle for differentiation is perhaps stronger than ever, with “interlopers” like bloggers, social media pundits, and other digital newcomers casting doubt on where the line should be drawn (Eldridge, 2017, 2019). To expel deviant actors and protect the autonomy of the journalistic field, journalists make distinctions between their norms or practices of new entrants. The incumbency of institutionalized traditions and practices of established news organizations forms much of the doxa of the journalistic field and resists the innovation of newcomers (Tandoc & Foo, 2018, p. 42).

Application of norms varies by context. In the case of small publications, such as the rural newspapers, the limitations of operating a newspaper with few staff in a small community can challenge the actualization of journalistic doxa. For example, an “ethical wall” is supposed to protect the autonomy of the editorial actions of a newspaper from the interference of managers (Broersma & Singer, 2021; Soloski, 1989). Despite the current economic pressures on the newspaper business, the ethical wall remains a journalistic ideal (Coddington, 2015). However, in the case of a small newspaper, such a wall may be difficult to achieve, pushing rural newspapers even closer to the heteronomous economic pole (Hess & Waller, 2016). Indeed, separation may be outright impossible when the staff contracts to the point where there is no longer a dedicated publisher distinct from editorial staff (Nagel & Broersma, 2024). Other norms, too, are difficult to actualize in smaller publications in rural settings (Smith, 2018). For example, a local newspaper may perform “boosterism,” directly portraying their town in a positive light and serving a hybrid role as town promoter, community member, and journalist. Boosterism is an idea often repugnant to major-market journalists yet common in local journalism (Hess & Waller, 2012, 2016; Olsen, 2021; Perreault et al., 2023). The practice, listed a century ago by Allen (1928) as one of the most important traits of a rural journalist, is still engrained in rural journalism, but can be viewed through an urban-normative lens as problematic, divisive, unethical, and lacking objectivity (Gutsche, 2015).

Where, then, do rural newspapers look for ethical guidance specific to their practices? The Canadian National NewsMedia Council (2025) provides links to ethical resources on its website, but nothing specific to rural newspapers or even smaller publications. An indicator of the state of journalistic boundaries in rural Canada comes from the industry itself. News Media Canada (a separate organization from the aforementioned NewsMedia Council) is the national industry association for newspapers and is responsible for disbursing funding for the Local Journalism Initiative, a federal government program that has supported local reporter positions at hundreds of rural Canadian newspapers. One requirement of the program is that applicant newspapers must have an editorial policy outlining the publication’s ethics. However, the application does not specify what that editorial policy must contain or espouse. On the program website, one of the “Frequently Asked Questions” is “I don’t have an editorial policy” (News Media Canada, 2025). Helpfully, the website provides a complete, generic editorial policy that newspapers can include as part of their grant application, with a suggestion to tailor it to one’s specific publication. While one could view this boilerplate document as a form of maintenance and policing of the dominant doxa and habitus in the field, it is an open question whether policies initially adopted for a grant application would be implemented in the day-to-day operation of a publication. Regardless, formal ethical guidelines don’t seem prominent in Canadian rural journalism.

Considering the evidence in scholarly and popular literature for rural journalism to be considered differently than other forms of journalism, this study poses the following research questions:

**RQ1:** How do rural journalists’ content creation practices differ from practices in metropolitan journalism, as documented in the literature?
**RQ2:** How do rural journalists’ relationships with the community differ from such relationships in metropolitan journalism?
**RQ3:** How do rural journalists’ business practices differ from those in metropolitan journalism?

## Background and Method

The research was conducted in the Canadian province of Alberta, with a population of approximately 4.2 million. The majority of the province’s population lives in an urban/suburban corridor between Calgary and Edmonton, but there are approximately 1.5 million residents who live in smaller towns and rural areas outside of this corridor. Rural areas in Alberta can be very remote. Neighboring towns are often separated from each other by dozens (and in some cases, hundreds) of kilometers with only farmland or wilderness between them. Population densities in rural areas of the province vary from one person per 10 km^2^ up to about 50 people per 10 km^2^. The remoteness, coupled with the significant geographic separation between towns, makes Alberta an ideal place to study local media because there is very little overlap between local publications. Few rural Albertans, especially in remote areas, read the next closest town’s newspaper.

Newspapers are the predominant form of journalism in rural Alberta. Few rural radio stations devote significant resources toward journalism, and there are almost no rural television newscasts. While there are news websites, they generally exist as former newspapers that have moved to online-only distribution. Sometimes this move online is a pretense for the creation of a “ghost newspaper”—a title owned by a newspaper chain that in fact just runs wire copy on the website with no local reporting whatsoever (Abernathy, 2020). Chain ownership accounts for approximately 57% of Alberta newspapers, with the balance of newspapers published by independent owners. In total, there are now just over 100 newspapers in Alberta (including dailies and weeklies), but since 2008, there have been at least 57 closures of local media outlets in the province (Lindgren et al., 2018, 2020). Most rural towns in Alberta now have only a single publication, and an increasing trend (particularly among chain-owned newspapers) is regionalization: individual town newspapers are closed, and the staff and resources are aggregated to create a regional publication covering several towns. This is generally done as a cost-saving measure, often involving reductions in staff and service.

This study examines rural Alberta newspapers only. In a purposive sampling method, four communities with local newspapers were selected—newspapers dedicated solely to a single community as opposed to regional titles with coverage of several towns. The communities were selected to represent a variety of community and business environments (see Table 2). To create as diverse a sample as possible, geographic and economic factors, as well as the nature of the newspaper, were considered.

**Table 2.** Participant Communities.

Location	Industry	Number of residents	Newspaper ownership	Reporter staffing
Southern Alberta	Agriculture	2,000 and gradually increasing	Chain-owned	One reporter
Northern Alberta	Mixed economy	3,000, fluctuates according to economic conditions	Independently owned	One reporter
Central Alberta	Resource extraction	3,000 and declining	Independently owned	One reporter
Central Alberta	Mixed economy	1,500 and stable	Independently owned	One reporter

In each community, initial contact was made with the newspaper, and then in-person community visits were conducted. A total of 8 days were spent in one community, 4 days in each of two communities, and a single day in a fourth community. The variance in visit duration was due to practical challenges of conducting research in rural communities far from urban environments. During the visits, which occurred in the summer of 2023, the researcher followed the daily routines of newspaper staff and community members in a participant-observation methodology, culminating in formal interviews with journalists and community members. Written field notes were taken during times of participant observation, and these field notes were annotated and expanded at the end of each day to accurately record and develop the events of the day (Emerson et al., 2011). Formal semi-structured interviews (*N* = 23) were organized according to an interview guide including themes of role definition, practice, impact of journalism, and views on various forms of community information (including newspapers and social media). Separate guides were used for journalists and for community members. During interviews, participants were given the choice to be voice-recorded, with five participants agreeing. For those that were not recorded, detailed written notes were taken during the interview.

A total of eight newspaper staffers and ex-staffers and fifteen community members were interviewed. Job titles of interviewees included journalist, reporter, newspaper owner, publisher, production manager, town administrative officer, tourism information worker, library technician, local historian, and several others. In one community, an interview was conducted with the owner of a defunct newspaper (in addition to the interviews conducted with participants from four newspapers that were operating at the time of the research).

The study was approved by a Canadian research ethics board (certificate #100127). Consent was obtained from interviewees, who were all afforded anonymity so that they could talk freely. The results and quotes included below are not attributed to specific communities or publications. In many cases, news workers were aware that their practices were unconventional and were concerned that their publication or community would be identified and cast in a bad light.

During the analysis, interview data and participant observation notes were subjected to a thematic analysis using a grounded theory approach. Thematic analysis involves examining a body of research data for themes and subthemes—motifs that recur within the dataset (Bryman, 2012, p. 578). These themes and subthemes were developed both inductively (through the familiarization process) and deductively (as the data were examined for the inductive themes) through an iterative process. While several practices were observed in more than one community, the methodology of this research did not allow for direct comparison between communities. This research sought to analyze the presence of rural-specific practices, and future research will contrast practices in individual communities.

## Findings

In our analysis, 12 counter-doxa practices of rural journalists emerged, which are outlined below, according to the research questions.

### RQ1: Creating and Distributing Content

Six content creation practices were found that differ from practices in metropolitan journalism as documented in the literature. First, journalists *acted as community boosters*. Pressure to perform this role is not new to local journalism (Hess & Waller, 2016). Small newspapers view their audience as local, which means that boosterism is a display of support to community members, not to outsiders. One newspaper owner explained why the paper gave prominence to local businesses over businesses in neighboring towns. Beyond the first being more relevant to local readers, the owner felt a moral obligation to advance local interests:

*Part of being in the community is sticking up for the community. I want to be seen as a community supporter, not making the community look bad. No one wants to read that.*

Boosterism can occur outside of news coverage, in the daily business operations of a newspaper. At one newspaper during COVID-19, boosterism included providing no-strings-attached free advertising for community businesses.

Second, *story vetting and image manipulation* emerged as divergent practices. Traditional journalistic practices at rural publications are sometimes flexed because of the realities of day-to-day news coverage in a small town. Community expectations collide with the impetus to provide coverage of important events, leaving the rural reporter walking a fine line. An example is allowing sources to preview an entire story prior to publication, a journalistic practice uncommon in North American journalism (Kent, 2020). One community journalist talked about breaking this norm.

*I’m not here to do a big expose on someone. But they can be nervous talking to me. Sometimes it’s the only way I can do it to agree to let them see the story before it’s printed. I know you’re not supposed to do that.*

At a local sporting event, another reporter considered their options when covering a sports team with an objectionable word in the team’s name. The reporter mused whether the name on the jerseys should be altered in the photograph because “people will be upset if their kids see that word.”

Third, rural journalists indicated that they strive for *avoiding controversy*. Several participants spoke of a desire not to embroil the newspaper in controversy—a significant difference from what many perceive the role of journalism to be. A primary motivation seemed to be avoiding polarizing attitudes toward the newspaper and to avoid creating divisions in the community (particularly after COVID-19).

The lengths to which newspapers went to avoid creating controversy were varied. One newspaper owner of a now-defunct newspaper said that they didn’t want to create divisiveness in the community, and relied on neutral, unbiased coverage.

*No one else covers the council meetings. I did that. People need to know what’s going on. I just presented what happened at the meeting. I didn’t do anything more. But sometimes people took offense that I printed those things. Now, the minutes are the only record of what the council does.*

At the other end of the spectrum is a unique publication that has completely eschewed traditional journalistic practice in terms of story idea generation. The publication will not cover any kind of news that they view as “negative” or “controversial.” Instead, it primarily publishes positive feature content.

*I want to make people in the community feel good about themselves and bring them together. People find out about the sad and polarizing things on Facebook. I don’t need to publish it in the paper.*

However, that publisher softened their stance when questioned about specific issues. They related a tale of a resource development community consultation that many residents didn’t know about. They printed information about the event in the newspaper but didn’t take a position on the issue.

*It’s really important. One way or another, it will change this community a lot. We just told people about the consultation and that they should make sure their voice was heard. But we didn’t tell them what to say.*

One of the ways that publications avoid controversy is by focusing on the creation of feature stories instead of hard news or opinion writing. The only reporter at one newspaper said:

*For me, it’s primarily feature content. People like reading that. They like to see their name in the paper on a feel-good story. I’m not here to make people feel bad.*

Fourth, rural journalists engaged in concerted efforts to maximize the numbers of community members in content. Particularly in rural communities, people like to see their names in the paper. One way of maximizing the number of residents who made it into the newspaper is to increase the number of people quoted. A reporter said that they try to interview as many people as they can for each story, even if their views are not substantially different. Reporters also spoke of the importance of including pictures of as many people as possible.

*I’d rather run a photo of a bunch of people lined up against a wall*—*the execution-at-dawn style of photo*—*than I would run a really good photo of a single person.*

Another way to incorporate community voice into a newspaper is to run submitted content verbatim. Even when poorly written, it is viewed as essential to making residents feel that the paper represents them as a community. One reporter spoke about running content from a youth agricultural club:

*I run those [community organization name redacted] submissions from the kids as-is. I mean, I edit the spelling mistakes but that’s about it. It’s a big thrill for a kid to see the story they’ve written published in the paper.*

Fifth, we found a regressive attitude toward online news and a preference for print. Rural newspapers are reluctant to divert significant resources toward publishing content on social media and are not even convinced of the benefits of posting news to websites. All rural newspapers in this study place primary emphasis on the printed edition. However, three of the four newspapers had websites of varying complexity. In the case of the paper with no website, some content was posted to Facebook. Despite this variety of online approaches, there was a general distaste for posting news online. One publisher explained:

*Being in print is what sets us apart from social media. We can create a physical thing*—*social media is ephemeral. When we print something, we can’t delete it if it’s wrong. And people know that. It’s why they trust us.*

Readers, too, relate differently to print—or at least reporters think they do. A local journalist related how local businesses and community members treasure being in the paper:

*It’s amazing to walk into a business and see your own work [as a reporter] framed and on the wall. I don’t understand how being mentioned on a social media post compares to that.*

Another driver of the preference for print is economic pragmatism. One publisher explained that readers are reluctant to pay for what they can get online for free, and that monetizing website content is difficult. Another publisher described a marketing role for the print edition: the durable print product found around town serves an advertising function for the newspaper itself.

Sixth, rural journalism has a complicated relationship with social media. In small towns, Facebook is the dominant social media platform. Facebook is “where it’s at,” a tourism information worker said. “That’s how people find out what’s going on.” However, Facebook is often not used by young people in the community, nor do they commonly read the newspaper. One young worker at the local library explained,

*Young people use Snapchat or Instagram to talk to each other and stay up to date. They use Facebook to talk to, you know, the older people. . . I didn’t read the newspaper, but now that my partner is working as a local recreation director, she is in the paper more often. Now I read the paper to see her.*

Newspapers sometimes publish their content on social media, but it is often an afterthought. One newspaper has poorly updated Facebook and X accounts. There is no one but the local reporter to update them, and they were told by their publisher that it’s not as important as other work:

*I should probably do more on social media, but it’s hard to fit in between shooting, writing and proofing the flats. I do everything and social isn’t really a priority.*

However, reporters say that social media is an important source of story ideas. In the case of one newspaper, most story ideas originated as a post on the local community Facebook pages. Reporters were dispatched to follow up on the posts and create “proper” news stories based on them. Photos, as well, were frequently sourced from social media for events that reporters were unable to attend.

Correcting falsehoods found on social media pages was a duty identified by staff members at two participant newspapers. They view their publications as having an invigilative role. Expressing visible frustration, one reporter was so fed up with the futility of correcting social media posts, they didn’t even want to talk about Facebook in the interview. Another newspaper publisher said:

*We’re different from social media because we have to print the truth, and people know that. It’s why they trust us. But when we’re gone, no one will be able to do that.*

### RQ2: Relationships With the Community

We found two distinctive practices related to how rural journalists’ relationships with the community differ from such relationships in metropolitan journalism. First, journalist *acted as politicians*. As mentioned in the introduction, in one small community, the local paper has a history of being involved in politics. Currently, the reporter for the newspaper is a town councilor, and in the past, the publisher and owner of the same newspaper has also been on the town council. Explaining their commitment to community service, the local reporter didn’t see a conflict of interest in the two roles:

*The stories I write about the council just talk about what went on at the meetings. I don’t get into the political stuff.*

But the publisher described how conflicts arose in the community during their previous work as a local politician:

*I tried so hard to get the town to invest in economic development. Just look at what [neighbouring community] did. But people just got resentful. They liked low taxes more than revitalization.*

Even so, the publisher did not identify any conflicts that arose through the actions of their newspaper. They perceived the disagreements as limited to their role at the time as a member of council.

Mingling of roles occurred in other participant communities as well, usually out of economic exigency due to low-paying newspaper positions. While not as dramatic as the reporter-politician, other combinations observed were reporter-independent vlogger; reporter-independent filmmaker; and publisher-civil servant.

Secondly, the roles of being a journalist and being a community member merged. In rural communities, a journalist must be a community member as well as an objective news gatherer. While their day job may be to create news content, journalists usually reside in the community they cover. Long a part of local journalism, reporters must eat, shop, and socialize with the residents they write stories about (Hess & Waller, 2016). Separating the role of journalist and resident can be almost impossible. One reporter related being pitched on story ideas as they were shopping their groceries.

Journalists are generally valued members of the community—and are sought after to cover events. There is an expectation on the part of community residents that the journalist be present at community happenings, even when news gathering is not actively taking place. In the case of a community softball tournament, a reporter said:

*I’m not here to get a story per se. I’m here to hang out. I’m here to show face. Showing face is important to these people. . . I’ll get the results of the softball tournament from the town office tomorrow and write the story from that.*

By the end of the day, however, two community members had approached the reporter with story pitches. In one case, the reporter conducted an interview on the spot, and in the other case, an interview was scheduled for later in the week. With a big smile, the reporter said, “And that’s why I came today.”

At a police outreach BBQ event in another town, a reporter echoed these sentiments.

*I’m here to talk to people. I won’t interview the police officers now. I want to let them to do their thing, and I don’t want to get in the way. I’m here to be seen, to talk to people. I’ll do the interviews later in the week.*

Showing up at events and taking pictures is not enough to meet community expectations, however. The presence of a reporter means that community members expect that a story (or at least photos) from the event will be published in the paper. One journalist related a formative story of a broken promise in their childhood and vowed to not repeat it in their current reporting role:

*When I was a kid, a reporter came to our teddy bear picnic event and took pictures and interviewed me. I was so excited to be in the paper, but a story on the event was never published. I was crushed. I’m never going to do that to this community.*

Despite a general decline in the fortunes of rural newspapers, community engagement can yield benefits for small-town newspapers. One local journalist was pleased with how outreach has doubled the page count in the paper they work for:

*My predecessor didn’t show face in the community. But in the year since I’ve been here, I show face. And people like that and buy ads. We’ve gone from a paper of eight pages a week to twelve, and sometimes sixteen. And that’s because of me.*

However, attending events when “off the clock” can be trying when personal life intersects with reporting work. A reporter showing up at an event (as a private individual) and not writing a story about it can be disappointing for community members. One newspaper publisher, who no longer routinely writes stories themselves, described the challenge of participating in private social events, but feeling social pressure to provide coverage even when they are not working.

*When I go to social events that my reporter is not at, people always ask if I’m going to write a story on it, even though I haven’t done any reporting for years. They see me there and they think there’s going to be a story in the paper. Then I write the story but don’t put my byline on it.*

### RQ3: Business Practices

We found four practices that were related to business practices that diverged from those in metropolitan journalism. First, *advertisements were made contingent on news coverage*. Engrained in the doxa of North American journalism is the separation between editorial content and advertising. But grinding business realities have altered this practice in some cases. One newspaper publisher described how they won’t do what they term “preview stories”—stories to announce an upcoming event or new business opening—without the organization buying an ad in the paper. Of special note is that the preview story (an advancer) is not labeled as an advertorial in the newspaper.

*It used to be that businesses automatically bought an ad in the paper when we did a preview story. But they don’t do it anymore, and we can’t afford to do those kinds of stories for free.*

The reporter at this publication shrugged off the policy—while admitting that it was unconventional.

*It’s not really what they teach you in j-school [journalism school] but I understand where [name of the publisher redacted] is coming from. I’m his employee*—*he’s my customer. And I do things the way he wants them done.*

Second, rural journalists have *unconventional relationships with local governments*. In the wake of financial downturn for local newspapers, the search for alternative funding sources has been an important topic for small-town newspaper owners. In opposite ends of the province, two newspapers have developed similar arrangements with the local county governments. The county government buys a newspaper subscription for each county resident. In exchange, the newspaper provides the local government with significant space in the paper each week to print county news. The content is not created by the newspaper journalists but comes straight from the county office. While it is not journalistic, it doesn’t always look like an advertisement either.

*That deal saved the paper. It pays for our printing and distribution costs, so we just need to run the office and pay the reporter.*

A reporter at another newspaper responded to a question about declining readership at community newspapers:

*The deal with the county means that everyone gets our paper, so our readership is not declining. But I guess they don’t all read it. I think some people line their birdcages with it instead.*

Third, we encountered *owners who were funding their paper out of altruism*. Independent rural community newspaper publishers are sometimes extremely idealistic about their publications, keeping them going for a period after they cease to earn revenue. Sometimes, this period is years. One local publisher of a now-defunct title expressed regret that they kept it going for as long as they did—ultimately losing a significant sum of money. Another local publisher said their newspaper has been running at a loss for years.

*We are in debt and need to change things. My reporter says that he wants to be the last reporter at the [publication name redacted]. I don’t know how much longer I can keep it going. We never had a two-consecutive-year decrease in revenue until 2014. But then things changed and it’s down each year.*

Altruism has its limits: One newspaper publisher describes the difficult decision about beginning to charge for death announcements. At many newspapers, such notices have traditionally been paid for by the bereaved, but at this newspaper, it was a big change.

*We never used to charge for obits. We printed them as a service to the community. Now, we have to limit the length of them. It’s just too expensive for us to print them. We’ll do a certain amount for free and then we charge them if they want to write more. It’s hard to tell people that after we’ve done it for free for so long.*

Fourth, *value-based decisions were made ahead of publication viability*. Simultaneously with the erosion of the “ethical wall” at rural newspapers, as discussed earlier, publishers told of courageous decisions that threatened (and in one case, terminated) the viability of newspapers. This is an example of where rural newspapers have chosen to adhere to traditional journalistic doxa despite economic ramifications jeopardizing the entire publication.

The purchase of ads can be weaponized when local businesses attempt to influence newspaper coverage. A former newspaper publisher describes how a local business disagreed with the coverage the newspaper provided about a council meeting. The dispute led to a significant reduction in overall ad revenue for the paper, hurting its long-term viability. Ultimately, the newspaper closed after losing money for several years.

*They demanded that I retract the story, which I wouldn’t. Then they pulled their weekly full-page ad. That cost me a thousand dollars in ad revenue right away. That hurts and was definitely the start of the end.*

Another publisher related past challenges in holding local authorities to account, including being the victim of attempted arson at the newspaper offices after controversial content was brought to light. They were ultimately unable to obtain insurance reimbursement for the event but persisted in the investigative efforts after the loss.

## Discussion

When analyzing how rural journalists negotiate between the dominant metropolitan-based doxa and habitus of the field, and their position within rural communities, Carlson’s (2015, p. 9) conceptualization of journalistic boundaries offers a useful lens (see Table 3). Plotting the diverging practices found in rural journalism against Gieryn’s three components of boundary work (expansion, expulsion, and protection of autonomy) and three factors (participants, practices, and professionalism) exhibits just how widely rural community newspapers deviate from traditional norms and practices associated with print newspaper journalism.

**Table 3.** Forms of Boundary Work in Journalism With Examples from Alberta Rural Journalism.

	Participants	Practices	Professionalism
Expansion	Incorporating non-traditional journalists	Taking over new media practices as acceptable	Absorbing new media as acceptable journalism
	*Journalist as community booster*	*Story vetting and image manipulation*	*Not observed in this study*
	*Journalist as politician*	*Avoiding controversy*	
	*Journalist as community member*	*Concerted effort to maximize numbers of community members in content* *Advertisements contingent on news coverage* *Unconventional relationships with local government*	
Expulsion	Expelling deviant actors	Expelling deviant practices	Expelling deviant forms and values
	*Not observed in this study*	*Not observed in this study*	*Regressive attitude toward online news and a preference for print*
Protection of autonomy	Keeping out non-journalistic informational actors	Defense of the ability to define correct practices	Defense from non-professional outsiders
	*Complicated relationship with social media*	*Owners funding papers out of altruism*	*Value-based decisions ahead of publication viability*

*Source.* Adapted from Carlson (2015).

*Expansion* denotes ways that the actors in the journalism field seek to expand the definition of journalism. In this case, we see an expansion in the attitudes of journalistic actors to include dual-role journalists (such as politician-journalists) that are repugnant to the orthodoxy in the field. We also observe a more permissive attitude toward traditionally taboo journalistic practices. This includes, for example, allowing sources to vet stories prior to publication and payment for news coverage. In terms of professionalism, there seems to be no expansion of the field into new techniques, technologies, or media. Weekly rural community newspapers have resolutely “circled the wagons” around print and have not embraced websites or social media in the same way urban counterparts have.

When it comes to *expulsion*, we see no tendency of rural journalism to expel deviant actors or practices. With such a precarious existence, the field of journalism (at least in rural towns) is not likely to winnow deviance and further diminish the field. Carlson (2015) describes how new media often has been an expansionary area in the journalism field. However, rural newspapers buck this journalistic trend of embracing new technologies and instead focus on a printed product (Smith, 2018). For rural publishers in this study, the publication of a printed product seems to be a key boundary line between journalism and other types of digital community information. In other words, the print edition is an ostentatious display of professionalism.

Finally, in the case of *protection of autonomy*, we observe a resolute differentiation from social media actors. In fact, some rural newspapers see a key role in refuting misinformation and opinion posted on local social media pages. In terms of practices, it seems that rural journalists have come to terms with breaking journalistic norms when they view themselves as serving the local community. In what might be viewed as a “life before limb” approach, the sustainability of the publication becomes more important than adhering to abstract normative behavior.

Dissonance was observed in the final square in the matrix: the nexus of protection of autonomy and professionalism. Despite the earlier documentation of modified journalistic ethics surrounding the publication of editorial content contingent on advertising purchase, we see some rural publishers making ethics-based decisions on important issues even when it imperils the publication. This shows that despite the expansion of practice, there is still a sense of traditional journalistic professionalism at rural publications. However, these decisions seem to be made using publishers’ personal moral compass rather than guidance from editorial codes submitted for the Local Journalism Initiative grants. Not a single participant referred to a specific code of ethics when discussing ethical decision-making.

The themes, taken together, portray a situation where newspaper publishers feel a greater sense of duty to a local community and their publication’s role in that community than they do to journalistic ideals. They see themselves as community members first, and secondarily, their role within that community as a journalist. This means that they are willing to compromise journalistic norms when it means that they can continue serving the community. For example, they maintain a differentiation between the newspaper and other forms of media (social media, government newsletters, etc.), but they care less about maintaining consistency with extrinsic journalistic ideals developed in geographically distant organizations. And they defend themselves against actors that would try to alter or influence the work of the newspaper, but compromise conventional doxa on *their terms*, striking deals with local government to cover distribution costs, accepting payment for feature stories and advancers, and assuming additional interest-conflicting roles such as local political office. Journalists in this study also recognized that their actions did indeed run counter to urban journalistic practice but resolved such cognitive dissonance by justifying their actions based on context and lived experience. Rural journalism norms are not binary, nor are they consistent.

Rural journalists, then, do not simply diverge from metropolitan journalistic norms out of necessity; rather, they function as heterodox agents, actively reshaping the field to align with the realities of their communities. Their adaptations—whether through unconventional ethical compromises, political entanglement, or direct financial arrangements with local governments—represent more than survival strategies. They are intentional recalibrations of journalistic practice that challenge the boundaries of professional legitimacy while maintaining their role within the field. This heterodoxy is not a rejection of journalism’s core principles but a pragmatic response to structural constraints, demonstrating that field boundaries are not static but constantly negotiated. By understanding rural journalists as heterodox agents, we gain a more nuanced perspective on how journalism evolves in response to local pressures, reinforcing the need to study rural journalism as a distinct yet fluid subfield rather than a mere outlier.

Our analysis shows a repertoire of rural journalism practices that differ from those of the larger journalism field’s established set of norms—in the product that is created, the processes (including the ethics) that lead to the creation of that product, and in the relationships that newspapers and their staff have with the community they serve. In their deviation from established North American journalistic doxa, rural community newspapers have made both intentional and consequential choices that set them apart from most other journalistic operations.

In examining these results through the perspective of field theory—and more specifically, Bourdieu’s conceptualizations of capital, we see rural newspaper workers forgoing cultural and symbolic field capital to amass—or at least maintain—economic and social capital. For example, a decision to assume additional, non-journalistic roles within a community indicates that a journalist is sacrificing cultural field capital—that is, the doxa of traditional North American journalism—to economic pragmatism of having enough economic means to sustain themselves. Further, we see tension between social capital and cultural capital. When a journalist decides to allow a local source to preview a story before publication, they sacrifice cultural capital by not adhering to the traditional journalistic doxa. On the other hand, they amass social capital in the community by generating goodwill and reinforcing that they are a community member before they are a journalist. This has the effect of increasing trust because of a closer connection between the reporter and their community (Armstrong & VanDyke, 2025).

There has long existed in journalism a significant tension between heteronomous economic capital and autonomous cultural capital (Benson & Neveu, 2005). But particularly difficult decisions face rural newspapers trying to sustain themselves. For example, in deciding to write only features about businesses that also pay for ads, a newspaper publisher sacrifices cultural capital in exchange for economic capital. However, this decision is existential. If a local newspaper publisher is no longer able to generate revenue, the publication will no longer continue to exist. The choice is not a value judgment between sacred and profane; it is a question of life or limb. The dedication to maintaining some kind of journalism in a news desert community, as exemplified by the newspaper publisher in this study that has been producing the news at a financial loss for years, demands that sacrifices be made in adherence to journalistic norms. It is an idealistic decision, not a crass self-serving one.

## Conclusion

Rural journalists negotiate between metropolitan journalistic doxa and the expectations of their communities by selectively adapting norms and practices to suit their unique circumstances. Rather than simply replicating dominant journalistic conventions, they recalibrate their roles to balance professional ideals with the economic and social realities of rural life. This process of adaptation aligns rural journalism with the characteristics of a journalistic subfield—one that operates within the broader journalistic field while maintaining distinct practices and norms (Marchetti, 2005; Siapera & Spyridou, 2012). Subfields take their overall doxa from a parent field but are also shaped by external influences, complicating their boundaries (Hilgers & Mangez, 2014; Thomson, 2014). An example of such conflict in journalistic doxa is the economic arrangement for exclusive journalistic access to an event, a common practice in sports journalism but anathema to general journalistic ethics (Marchetti, 2005, p. 72). Following this logic, then, rural journalism is well described as a subfield of journalism, albeit a field with significant distance to the core North American journalistic doxa.

The existence of this subfield is a direct result of the challenges journalists face in negotiating between the dominant metropolitan-based doxa and habitus of the field and their position within rural communities. The subfield of rural journalism is a compromise—an adaptation of the doxa and habitus of the overarching journalistic field to the expectations and demands of practicing journalism in a small-town context. But negotiating between these two forces is not the only challenge of rural journalism. The recent economic precarity that has resulted in the closure of many rural newspapers raises another question: at what point is the subfield of rural journalism so sparsely populated and diverse in its doxa that it ceases to be a cohesive subfield?

Already, Ryfe (2012, p. 155) has described the overarching journalism field as unraveling because of divergent heteronomous forces leading to an increased diversity of organizational practice. This is exacerbated in the case of rural journalism, where a continuing divergence of doxa and habitus, combined with a significantly reduced number of members, challenges the cohesion of the subfield. Lack of cohesion in a field with agents no longer agreeing on “the definition of the stakes” can eventually spell “a catastrophic collapse of its autonomy” (Steinmetz, 2016, p. 102). And so, we can imagine a rural journalism subfield eventually so sparsely populated and distributed over such a vast geography that significant deviation from even rural journalistic norms can occur without a Gierynian expulsion of members from the subfield. Given an already significant deviation of rural journalistic practice from the norms of the overarching journalism field, a collapse of the subfield of rural journalism would be accompanied by an exit from the overarching journalism field as well, leaving a landscape of widely separated surviving publications.

However, the rural newspapers we studied are presently still well within the boundaries of journalism. Exploring its boundaries, Eldridge (2017) proposes that journalism is

a distinct field in society identified by its members’ expressed adherence to shared traits and roles, underpinning the transmission of new facts as information to a public in their interest; further defined by a provision of context and analysis, built on expertise. (p. 24)

This is an accurate description of all newspapers described in this article. Certainly, members of the rural journalism subfield presently distinguish themselves from quasi-journalistic interlopers such as Facebook groups, indicating a sufficient degree of cohesion currently remains in the subfield. Not explored in this paper is the impact that interlopers might have on North American journalism objectivity norms, and whether such norms extend to the subfield of rural journalism in a unified fashion. Plenty of the practices documented in the findings are anathema to objectivity (the practice of boosterism among the most obvious), but on the other hand, objectivity is sometimes less important in non-North American contexts, and certainly less prominent in interloper media. Such objectivity (or lack thereof) may be further challenged by the increasingly divided and hyperpartisan political environment.

As with any study, this research has limitations. It was limited to diverse communities within a single Canadian province and conducted through a lens of North American journalism, which may differ greatly from rural contexts globally. Further research in additional Canadian regions and internationally might reveal additional insights or provide interesting contrasts between Albertan and other forms of rural journalism. In addition, although four operating rural newspapers were included in the research, research involving additional papers would yield results that might be more broadly applied.

Moreover, though this paper analyzes deviation from traditional North American journalistic doxa, it does not analyze differences between rural newspaper outlets. We have, however, presented evidence of the existence of rural journalism as a subfield, which will help demarcate future research and provide a framework for further inquiries. While elements of cohesion exist within the data, each of the four newspapers discussed also operates differently from the other. Additional work is needed to examine practices at individual rural newspapers for evidence of cohesion and diversity within the rural journalistic subfield, as well as changes that may be happening over time.

Journalism is not something that “is” but the product of continuous negotiations. A field is composed of forces that are always in flux. In the case of rural publications, these forces continue to reduce the population of the field. The remaining members of the rural journalism subfield must continue to adjust their habitus and doxa to adapt to the changing circumstances, or they must exit the field. But regardless of membership in a subfield or adherence to doxa, the fact remains: despite some deviance from journalistic norms, remaining rural journalists are also community members, full of idealism and dedicated to serving their fellow residents even when it comes with personal costs. We believe that the identification of rural journalism as a distinct subfield will aid both scholars and practitioners in developing a more complete understanding of rural journalism—a conceptualization that may lead to increased awareness of the importance of these important community members.
